# Atypical eclampsia and postpartum status epilepticus

**DOI:** 10.11604/pamj.2015.20.17.5831

**Published:** 2015-01-07

**Authors:** Zeynep Ozcan Dag, Yuksel Isik, Yakup Turkel, Murat Alpua, Yavuz Simsek

**Affiliations:** 1 Department of Obstetrics and Gynecology, Kirikkale University, Faculty of Medicine, Kirikkale, Turkey; 2 Department of Neurology, Kirikkale University, Faculty of Medicine, Kirikkale, Turkey

**Keywords:** Eclampsia, preeclampsia, status epilepticus

## Abstract

Preeclampsia is an entity that may present from 20th week of gestation up to 48 hours postpartum and is associated with hypertension and proteinuria. Eclampsia is emergence of convulsions pre-eclampsia in pregnant women with signs and symptoms. Recent studies showed that in some women, preeclampsia and even eclampsia may occur without hypertension or proteinuria. Here, we present a case of 26 years old women who had an uneventful pregnancy until 30 weeks' of gestation. She had only proteinuria in laboratory tests and was diagnosed as status epilepticus in early postpartum period. Preeclampsia and eclampsia is related with serious fetal and maternal morbidity and mortality and may present with atypical course. The awareness of atypical cases of preeclampsia enhances early diagnosis and management which are critical to avoid feto-maternal complications.

## Introduction

Preeclampsia is characterized with hypertension and proteinuria during pregnancy, and can have serious consequences about maternal and fetal health. The incidence of preeclampsia in developing countries is 12% and it also constitutes a significant proportion of maternal mortality in developed countries. Complications such as acute pulmonary edema, intracranial hemorrhage and eclampsia can also be negative fetomaternal consequences [1, 2]. Fetal outcomes of preeclampsia also include prematurity, intrauterine growth retardation, increased neonatal mortality, cardiovascular and metabolic disorders later in life and neurological disability [3]. Eclampsia is emergence of convulsions pre-eclampsia in pregnant women with signs and symptoms. In recent years, atypical cases showing atypical course have been reported [4]. The characteristics of these cases was reported as eclampsia in the absence of hypertension and proteinuria, a partial seizure following eclampsia with antecedent proteinuria without hypertension, a case presenting with fetal distress, without hypertension and a case with unusually rapid progression and massive proteinuria that was unresponsive to therapy. Here, we aimed to present a case that had no signs of pre-eclampsia during pregnancy follow-up, except for proteinuria and developed status epilepticus in the postpartum period.

## Patient and observation

**Figure 1:**
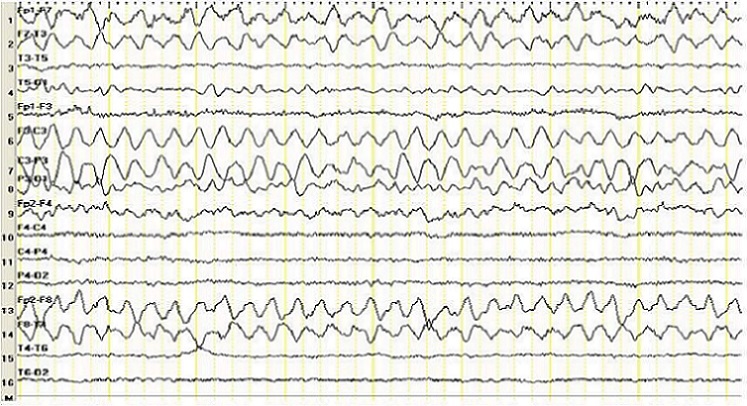
High amplitude teta slow wave activity in bilateral frontal regions

A 26 years-old nulligravida was presented with regular contractions at 30 weeks gestation. On admission, systemic blood pressure was normal. There was no symptoms at that time such as headaches or visual disturbances and all her prenatal visits had been normal, including blood pressure (BP), which was recorded as 120/80 to 110/70 mmHg. There was no prodrome of hypertensive disease and no laboratory abnormality, including platelet count, liver enzymes, LDH, electrolytes, and glucose, although proteinuria (3 + ) on dipstick was noticed on admission. There was no preeclampsia or other hypertensive disorder in previous pregnancy. There were no co-morbid conditions in the patient such as history of preexisting hypertension, diabetes or kidney disease. In the delivery room, she developed generalized tonic-clonic convulsions lasting 3 min, despite being normotensive. MgSO4 was given (loading dose of 46 g over 1520 min, followed by a maintenance dose of 2 g/h as a continuous intravenous infusion). As intrapartum fetal heart rate recordings revealed poor variability, an emergency cesarean delivery was performed. In the postoperative period within two hours of admission, second generalized tonic-clonic seizure was observed. Due to ongoing seizure, 0.1 mg/kg intravenous bolus diazepam was administered slowly. In the post-ictal period, her BP raised abruptly to 150/100 to 140/100 mmHg. Her BP continued high for two days, ranging between 160/100 and 140/90 mmHg and normalized on postpartum day 2, with 870 mg/dL proteinuria in the 24 h urine collected postpartum. The patient was consulted to the Neurology department. She had no focal neurological deficits. In order to rule out intracranial hemorrhage, brain tomography (CT) was obtained. It revealed no pathological findings. Due to recurrent seizures and unconsciousness, the patient was accepted as status epilepticus and phenytoin infusion of 20 mg/kg loading dose was initiated. Maintenance dose of 8 mg/kg phenytoin was infused. During the observation after 10 hours of admission, speech arrest and contralateral dystonic posturing and unconsciousness, a partial seizure, has been detected and 0.1 mg/kg intravenous bolus diazepam was administered slowly. After 16 hours of admission focal seizures which was shorter than previous ones ended without any medications. Electroencephalography (EEG) revealed 4 Hz frequency high amplitude waveforms in bilateral frontal regions at 15th hours of postpartum period (Figure 1). On the 3rd day of admission, control EEG was reported as normal. Phenytoin 300 mg/day was continued. She was discharged on the 7^th^ postoperative day.

## Discussion

New onset of hypertension and proteinuria during pregnancy is called preeclampsia. The diagnosis is easy in the presence of the classical findings, therefore fetal and maternal morbidity and mortality are significantly reduced. If preeclamptic patients present with atypical course, the treatment may be delayed and more serious statements such as eclampsia develops. In our patient, routine biochemical tests, coagulation studies and doppler uterine artery studies in early pregnancy were all normal. Proteinuria was found in the urine analysis during control visits but blood pressure was in normal limits. In this respect, our patient showed an atypical course as in previous reports [4–7]. Our patient did not have any complaints such as headache, blurred vision, and epigastric pain which are seen in preeclampsia cases. In the literature, 80% of eclampsia cases arise during prenatal period and childbirth. Eclampsia has emerged in the postpartum period in a few cases [8, 9]. In our case, the first seizure occurred before delivery. Status epilepticus is defined as one continuous, unremitting seizure lasting longer than five minutes, or recurrent seizures without regaining consciousness between seizures for more than five minutes [10]. Our patient who did not have epilepsy had her first seizure during the follow-up in hospital. Despite magnesium sulfate and antiepileptic treatment she was diagnosed as status epilepticus. Partial epilepsy such as frontal lobe epilepsy may not lead to loss of consciousness. Sometimes partial onset seizures can progress to generalized seizures. The clinical and EEG features of our patient were considered as eclampsia with partial onset seizure. Partial seizures may be overlooked because they are not obvious generally and than eclampsia diagnosis may be delayed.

## Conclusion

Preeclampsia and eclampsia which are important causes of maternal and fetal mortality may have atypical features in terms of clinical and laboratory findings. As in cases of proteinuria in the form of seemingly innocent isolated cases, atypical cases must be kept in mind in the differential diagnosis of preeclampsia so that this may have an important role in reducing maternal and infant mortality.
